# Clinical Applications of Anterior Segment Optical Coherence Tomography

**DOI:** 10.1155/2015/605729

**Published:** 2015-03-03

**Authors:** Su-Ho Lim

**Affiliations:** ^1^Department of Ophthalmology, Yeungnam University College of Medicine, Daegu, Republic of Korea; ^2^Department of Ophthalmology, Daegu Veterans Health Service Medical Center, 60 Wolgok-ro, Dalseo-Gu, Daegu 704-802, Republic of Korea

## Abstract

Anterior segment optical coherence tomography (AS-OCT) was recently developed and has become a crucial tool in clinical practice. AS-OCT is a noncontact imaging device that provides the detailed structure of the anterior part of the eyes. In this review, the author will discuss the various clinical applications of AS-OCT, such as the normal findings, tear meniscus measurement, ocular surface disease (e.g., pterygium, pinguecula, and scleromalacia), architectural analysis after cataract surgery, post-LASIK keratectasia, Descemet's membrane detachment, evaluation of corneal graft after keratoplasty, corneal deposits (corneal dystrophies and corneal verticillata), keratitis, anterior segment tumors, and glaucoma evaluation (angle assessment, morphological analysis of the filtering bleb after trabeculectomy, or glaucoma drainage device implantation surgery). The author also presents some interesting cases demonstrated via AS-OCT.

## 1. Introduction

Optical coherence tomography (OCT) is a noncontact optical device that provides cross-sectional images and quantitative analysis of the ocular tissues, mainly the posterior segment [1]. In 1994, Izatt et al. [2] presented the first report of the OCT image of the corneal and anterior segment. Anterior-segment OCT (AS-OCT) has become a crucial tool in clinical practice. In this review, the author discussed the various clinical applications of AS-OCT and its limitations.

## 2. Devices and Normal Findings

Anterior-segment OCT systems are categorized by wavelength of light sources; dedicated systems using 1310 nm (Zeiss Visante, Heidelberg SL-OCT, Tomey CASIA, etc.) and systems converted from a retinal scanner using 830 nm (Optovue RTvue, Optovue iVue, Zeiss Cirrus, Heidelberg Spectralis, etc.) [3]. Due to the different light sources, there are some differences between the two groups. A shorter-wavelength (830 nm, near infrared) system provides a higher axial resolution, but its imaging depth is limited. On the contrary, a longer-wavelength system provides deeper penetration, and a 1310 nm wavelength is strongly absorbed by water in ocular media, and as such, a small amount of the light reaches the retina.

**Figure 1:**
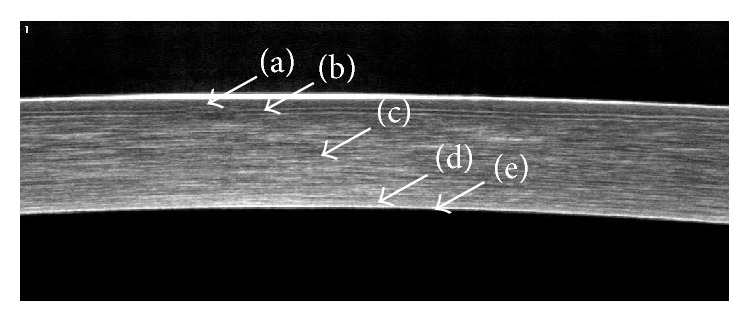
Horizontal OCT section of the normal cornea: epithelium (a), Bowman's layer (b), corneal stroma layer (c), Descemet's membrane (d), and endothelium (e).

Figure 1 shows the horizontal OCT section of the normal cornea using frame-averaged images. The ophthalmologist can distinguish a highly reflective tear film over epithelium (a), Bowman's layer (b), corneal stroma layer (c), Descemet's membrane (d), and endothelium (e).

## 3. Tear Meniscus Measurement

Tear film instability with potential damage of the ocular surface is an important concept in relation to the dry-eye syndrome [4]. Majority of the conventional tests, however, including the Schirmer test or staining, have the disadvantage of invasiveness, which influences the results [5]. Thus, various modalities have been investigated to evaluate the tear film, including AS-OCT.

**Figure 2:**
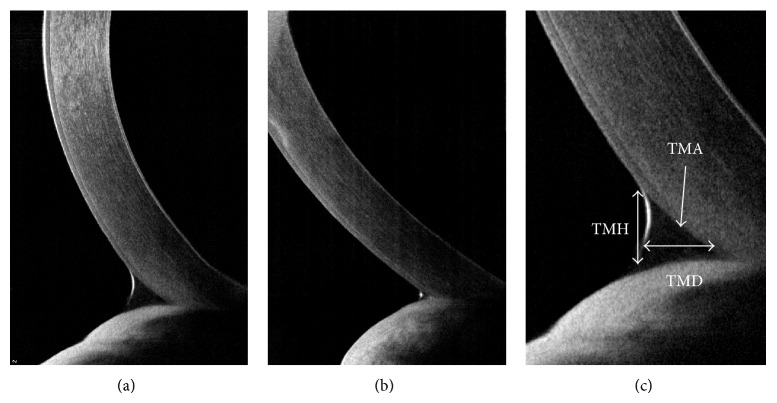
Tear meniscus measurement by AS-OCT. Normal (a), dry eye syndrome (b), three parameters that were usually measured (c); tear meniscus height (TMH), tear meniscus depth (TMD), and tear meniscus area (TMA).

Tear meniscus measurement via AS-OCT seems to be effective for the quantitative tear evaluation and diagnosis of the dry-eye syndrome or of patients with excessive tearing with punctal stenosis [5–7]. Tear meniscus measurement was recommended for taking an image immediately after blinking, and three parameters were usually measured: the tear meniscus height (TMH), tear meniscus depth (TMD), and tear meniscus area (TMA) [5] (Figure 2). Sizmaz et al. [8] reported that the tear meniscus height was lower in the patients with Grave's diseases compared to the normal control, which suggests that the tear function is significantly disturbed in Grave's diseases.

After the installation of an artificial tear [7] or punctal occlusion [9], AS-OCT was able to quantify a dramatic increase in tear meniscus. On the contrary, the tear meniscus height was decreased after the four-snip punctoplasty procedure [6] or dacryocystorhinostomy in the patients with epiphora [10]. In summary, OCT can be a valuable noninvasive and quick clinical tool for the evaluation of a tear film [9].

## 4. Pterygia, Pinguecula, and Scleromalacia after Surgery

**Figure 3:**
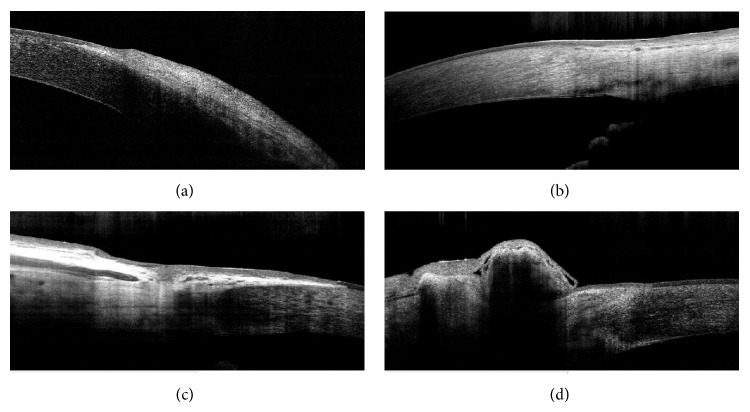
AS-OCT images of pterygium and pinguecula. Pterygium with corneal opacity (a), pterygium without corneal opacity (b), pseudopterygium (c), and pinguecula (d).

AS-OCT can provide high-resolution images of the anatomical relationship between the corneal tissues and pterygium and the pinguecula [11–13]. Soliman and Mohamed [11] reported that the primary pterygium revealed the elevation of the corneal epithelium by a wedge-shaped mass separating the epithelium from the underlying Bowman's membrane (Figures 3(a) and 3(b)). The image of the pseudopterygium showed that the overgrowing membrane was not really attached to the underlying cornea (Figure 3(c)). On the contrary, the OCT images of the pinguecula stopped at the limbal area (Figure 3(d)) [11]. The quantitative data obtained via AS-OCT also allow the accurate evaluation of the conjunctival changes over time after pterygium surgery with conjunctival autograft [14] and argon photocoagulation of the pinguecula [12].

**Figure 4:**
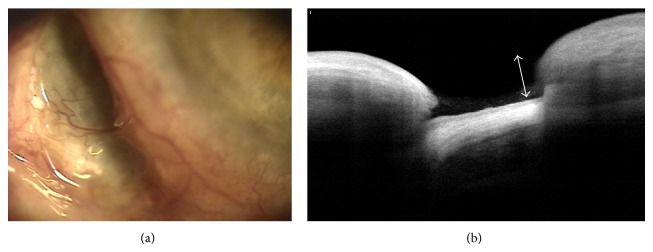
Anterior segment photography (a) and OCT image (b) of scleral thinning after surgery.

Besides the results of the previous studies [11–14], the interpretation of the AS-OCT may be helpful for predicting the residual corneal opacity after surgery and the difficulty during tissue dissection (Figures 3(a) and 3(c)). Moreover, the scleral thinning or scleromalacia after surgery can be repaired using a preserved scleral graft with or without amniotic-membrane transplantation [15]. Fortunately, with the aid of AS-OCT, the surgeon can consider the residual stromal-bed thickness and can estimate the graft thickness when planning surgery such as lamellar scleral graft or amniotic-membrane transplantation (Figure 4).

## 5. Architectural Analysis of Cataract Surgery: Cornea, Lens, and Biometry

**Figure 5:**
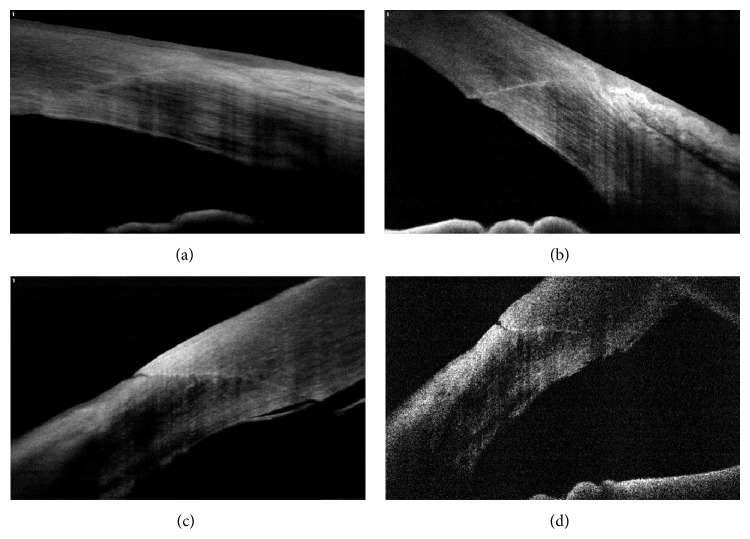
Architectural analysis of cataract surgery. Well apposed corneal wound (1 plane) (a), loss of cooptation with minimal endothelial misalignment (2 plane) (b), minimal Descemet's membrane detachment with epithelial gap (2 plane) (c), and loss of cooptation with endothelial gap (3 plane) (d).

AS-OCT was also used to image the clear corneal incision after cataract surgery [16–18]. On OCT, radial scans may be performed at the corneal incision site to analyze the following parameters: the curvilinear length (the total length between the internal and external wound openings), the linear length (the line between the internal and external wound openings), the angle between the corneal surface tangents, the architectural deformation, and the external depth of the incision [17]. In particular, the OCT image after cataract extraction can show the detailed wound architecture, including the plane of incision, Descemet's membrane detachment (DMD), endothelial misalignment, loss of coaptation, and endothelial or epithelium gaping (Figure 5).

With regard to the complications, the microaxial cataract surgery group had slightly fewer undesirable effects on the incision site compared to the biaxial group [16], and the femtosecond-laser-generated corneal incision had a significantly lower endothelial gaping and endothelial misalignment compared to keratome incision [18].

Recently, Nagy et al. [19] reported that AS-OCT imaging was able to detect the tissue changes within the lens after femtosecond laser capsulorhexis and nuclear fragmentation, and there was a case report demonstrating posterior capsular rent in posterior polar cataract detected via AS-OCT [20].* In vivo* three-dimensional (3D) biometry before and after cataract surgery was reported by Ortiz and colleagues [21].

In conclusion, AS-OCT provides sufficient information on the wound architecture and the biometric parameters, and thus surgeons can consider the structural stability of the cataract wound incision and can monitor the occurrence of complications.

## 6. Refractive Surgery and Ectatic Disorders

Keratectasia is a significant concern for refractive surgery. Therefore, many refractive surgeons have tried to minimize the incidence of post-LASIK ectasia. The ectasia risk factor score system provides a screening strategy to help minimize the risk and suggested that abnormal topography (forme fruste keratoconus), residual stromal-bed thickness, age, and preoperative corneal thickness are important factors [22]. The recommended residual stromal-bed thickness is 250–325 *μ*m [22, 23].

High-resolution OCT is helpful in the visualization of flap thickness, flap interface (flap-stroma relationship), and flap displacement [24]. Reinstein et al. [25] reported that the residual stromal thickness measured via OCT was thicker than that measured through very high-frequency ultrasound in many eyes with insufficient residual stromal thickness. Zhang et al. [26] demonstrated that flaps creased by femtosecond laser were more accurate, reproducible, and uniform compared to those creased by microkeratome. Timing for checking the LASIK flap thickness is also important. At one week, the surgically induced corneal changes were mostly resolved, and the interface can be easily seen via OCT. Thus, Li et al. [27] suggested that this time is best for measuring the flap thickness.

**Figure 6:**
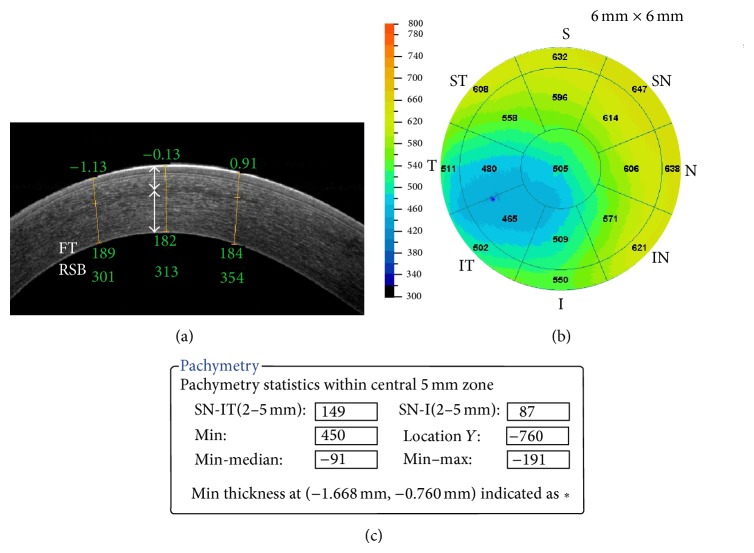
AS-OCT image of post-LASIK keratectasia. Horizontal OCT section demonstrating flap thickness (FT) and residual stromal bed thickness (RSB) (a), pachymetry map (b), and asymmetry parameters (c).

The detection of an ectatic change also has clinical pitfalls. Li et al. [28] provided several parameters for detecting the asymmetry and global or focal thinning, as follows: (1) I-S (the difference between the average thickness of the inferior octant and that of the superior octant) >31 *μ*m; (2) IT-SN (the difference between the inferotemporal octant and the superonasal octant) >48 *μ*m; (3) minimum <492 *μ*m; (4) minimum-maximum <−63 *μ*m; and (5) the thinnest region of the cornea is located outside the central 2 mm area. They suggested that one abnormal parameter provides suspect keratoconus, and two or more abnormal parameters provide a definite diagnosis (Figure 6).

The qualitative and quantitative evaluation of the cornea via AS-OCT before implantation of the intrastromal ring segment may offer safer surgery [29].

Corneal collagen cross-linking (CXL) has emerged as a promising technique to increase corneal stiffness and stabilize the ectatic corneal leading to inhibition of progression for keratoconus and postoperative LASIK ectasia [30]. A corneal stromal demarcation line indicates the transition zone (at a depth of approximately 300 *μ*m) between the cross-linked anterior corneal stroma and the untreated posterior stroma after CXL [30–33]. AS-OCT can visualize the demarcation line as hyperreflective line and evaluate the depth of the line which is correlated with the effective depth of the CXL treatment [31–33]. In recent comparative study, both confocal microscopy and AS-OCT have similar results in evaluating the depth of corneal demarcation line after CXL (confocal 306.2 *μ*m versus AS-OCT 300.7 *μ*m) [31]. Yam et al. measured demarcation line using AS-OCT and showed that it may decrease with the severity of ectasia and age [32]. And the mean depth measured by AS-OCT after CXL treatment is greater centrally in comparison to nasal and temporal depths (310.7 *μ*m centrally, 212.1 *μ*m nasally, and 218.0 *μ*m temporally) [33]. In summary, AS-OCT may also provide sufficient monitoring of the depth of the corneal demarcation after CXL as with confocal microscopy.

## 7. Assessment of Descemet's Membrane: Descemet's Membrane Detachment and Keratoplasty

**Figure 7:**
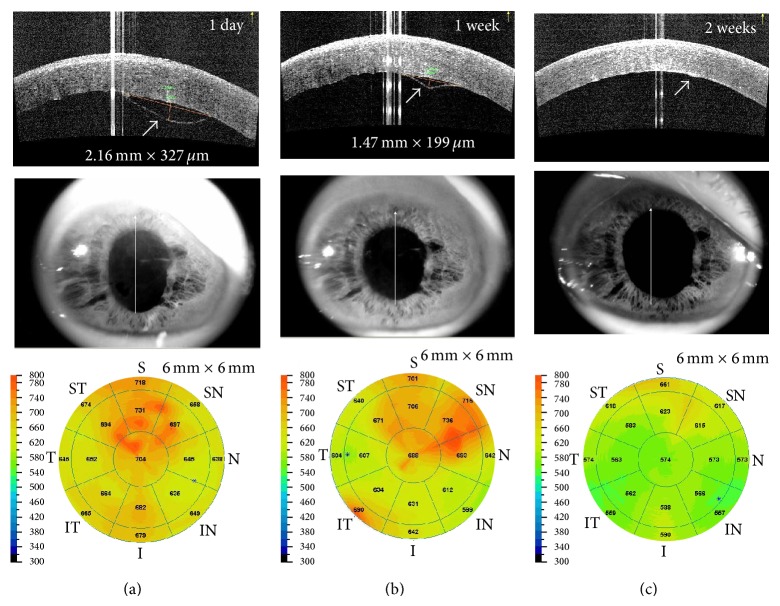
Spontaneous resolution of a detachment of Descemet's membrane following phacoemulsification. Optical coherence tomography (OCT) images at 1 day after cataract surgery (a) demonstrated superior planar type DMD with diffuse corneal edema. OCT images at postoperative 1 week (b) and 2 weeks (c) revealed spontaneous resolution of DMD without descemetopexy.

Descemet's membrane detachment (DMD) is considered a severe complication after intraocular surgery and trauma [34, 35]. Some DMDs, however, reattach spontaneously with a good prognosis, and a few corneas do not clear in spite of surgical treatment [31]. AS-OCT can demonstrate different statuses of the DMD, including planar/nonplanar, local/extensive detachment, and rupture [34–36]. AS-OCT is also a valuable tool for selecting the appropriate treatment and for monitoring the treatment outcomes when corneal edema is present [35] (Figure 7).

AS-OCT can also provide detailed information on the cornea after various keratoplasty operations, including penetrating keratoplasty (PKP), Descemet's membrane endothelial keratoplasty (DMEK), and Descemet's membrane stripping automated endothelial keratoplasty (DSAEK). The previous study [37] suggested that AS-OCT is an effective tool for the detection of an early graft detachment after DMEK, to determine if secondary intervention is indicated or is to be avoided. Yeh et al. [38] also reported that the one-hour AS-OCT scan showed the best predictive value of the six-month graft adherence status after DMEK.

The wound interface pattern can be shown by AS-OCT during DSAEK [36] or after PKP. Miyakoshi et al. [39] suggested that AS-OCT is useful for detecting the interface fluid between the host cornea and the graft during a DSAEK. Similarly, Sung and Yoon [40] showed that the alignment pattern of the wound interface after PKP differed according to the clinical diagnosis before surgery.

## 8. Explanatory Text, Figure 7

A 84-year-old male patient with benign prostate hypertrophy had underwent cataract surgery. During phacoemulsification, posterior capsular rupture was occurred because of severe intraoperative floppy iris syndrome. Thus, sulcus placement of single-piece acrylic IOL with anterior vitrectomy was performed.

On 1 day after surgery, slit-lamp examination revealed diffuse stromal and epithelial edema (central corneal thickness, CCT 704 *μ*m). Optical coherence tomography (OCT) demonstrated superior Descemet's membrane detachment (DMD, base × height, 2.16 mm × 327 *μ*m) and stromal thickening. The uncorrected visual acuity (UCVA) was 20/200 with no pinhole improvement and IOP was 14 mmHg. However, he deferred intracameral gas injection.

On postoperative 1 week, slit-lamp and OCT showed partial resolution of DMD (1.47 mm × 199 *μ*m). On two weeks after surgery, slit-lamp and OCT displayed uniform attachment of the DMD without any folds or gaps between Descemet's membrane and corneal stroma (CCT 574 *μ*m). The UCVA was improved to 20/32.

## 9. Corneal Deposits: Corneal Dystrophies and Corneal Verticillata (En-Face OCT)

**Figure 8:**
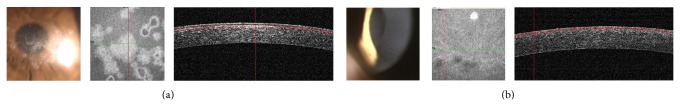
Anterior segment photography. En-face and horizontal OCT images of corneal deposits. Granular corneal dystrophy (a) and corneal verticillata (b).

When corneal opacities obscure the clinical differentiation between the anterior and deep infiltrates, OCT may determine the layers of the accumulation [41]. OCT measurement was reported to be highly repeatable: 2.1 *μ*m centrally and 1.2 *μ*m pericentrally [42]. Thus, AS-OCT provides useful information for the selection and planning depth of surgical procedures such as phototherapeutic keratectomy for removing corneal opacities with granular corneal dystrophy [43, 44] (Figure 8).

Other corneal deposits can also be demonstrated by AS-OCT. To the best of the authors' knowledge, there has been no case report with amiodarone-induced keratopathy demonstrated by AS-OCT. In a previous study with* in vivo* confocal microscopy [45], there were highly reflective and bright intracellular inclusions in the epithelial layers, and these findings were more evident within the basal cell layer in the patients with amiodarone keratopathy [44–46]. Some OCT machines can provide particular scan modes, such as en-face scan, which offers a new view of the different layers of tissue, like confocal microscopy [47]. In this patient, highly reflective and bright intracellular inclusions were observed mainly in the epithelial basal layer, and cornea verticillata was also detected easily in AS-OCT (en-face view) compared to the conventional slit lamp examination (Figure 8).

## 10. Keratitis

**Figure 9:**
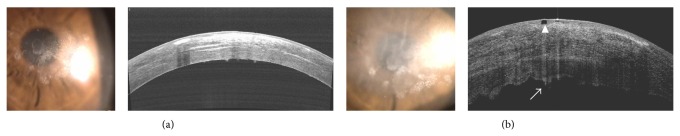
Herpetic keratitis with granular corneal dystrophy. In HSV keratitis with underlying granular corneal dystrophy, AS-OCT images (b) allowed the precise localization of microcystic edema (arrow head) and keratic precipitate (arrow) compared to baseline (a).

In clinical situations, the necrotic lesion and area of infiltration are not usually clear in microbial keratitis. Thus, it is easy for the cornea perforation and resection to become incomplete, and the recurrence of keratitis in severe cases needed surgical intervention [48]. Fortunately, the use of OCT allows the objective measurement of the corneal thickness and is an additional method for following microbial keratitis with greater accuracy compared to biomicroscopy alone [49]. Soliman et al. [50] reported that fungal keratitis grasped two unique patterns of early localized and diffuse necrotic stromal cystic spaces. Sun et al. [48] suggested that the removal of the necrotic tissue combined with conjunctival flap under the guidance of AS-OCT in the treatment of fungal keratitis is a safe and effective method. Similar to a previous report [49], in HSV keratitis with underlying granular corneal dystrophy, AS-OCT allowed the precise localization of microcystic edema and keratic precipitate in the subject patient (Figure 9).

## 11. Tumors

**Figure 10:**
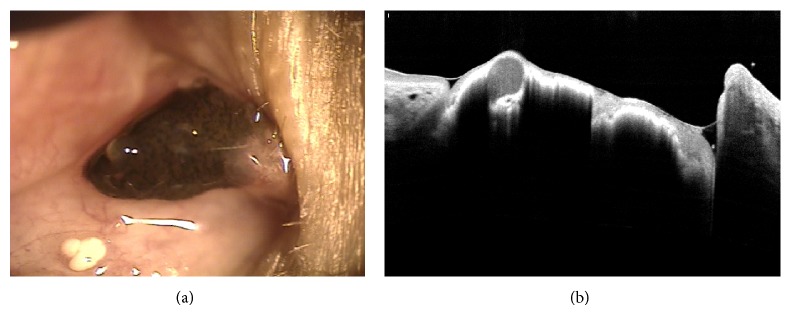
Anterior segment photography (a) and OCT image (b) of conjunctival tumor (nevus).

AS-OCT is a relatively reliable, convenient, and noncontact method for detecting and measuring anterior-segment tumors [51]. Image analysis [52] comparing UBM and AS-OCT with 200 patients, however, revealed the adequate visualization of all tumor margins (95% versus 40%), posterior tumor shadowing (5% versus 72%), and high overall image quality (80% versus 68%). UBM showed a better resolution for a pigmented tumor (66% versus 34%) and nonpigmented tumors (61% versus 39%). In another study comparing OCT to UBM in a nonpigmented iris tumor, the images of the anterior tumor surface and internal tumor heterogeneity were equivalent, but the posterior tumor surface was well defined in 54% of the OCT images versus 100% of the UBM images [53]. In conclusion, UBM showed the superior image quality and reproducibility of the anterior-segment tumor. Nevertheless, OCT is a noncontact, noninvasive technique that can be employed for supplementary examination in some selected nonpigmented tumors.  Figure 10 demonstrates that the OCT image showed a relative good anterior tumor surface, but the posterior tumor surface was not seen, like in the previous studies [52, 53].

## 12. Glaucoma: Angle Assessment and Laser Iridotomy

Gonioscopy is a gold-standard method for measuring the anterior chamber angle (ACA), but it is a subjective contact method with poor reproducibility and requires an experienced examination technique. On the contrary, AS-OCT can be performed easily, with relative good repeatability and reproducibility [54]. AS-OCT also provides several parameters about angle assessment [55–57]: ACA, angle opening distance (AOD 500 and 750 *μ*m), and trabecular-iris space area (TISA). One study suggests that AOD750 is the most useful angle measurement tool for identifying a narrow angle in AS-OCT images [58].

**Figure 11:**
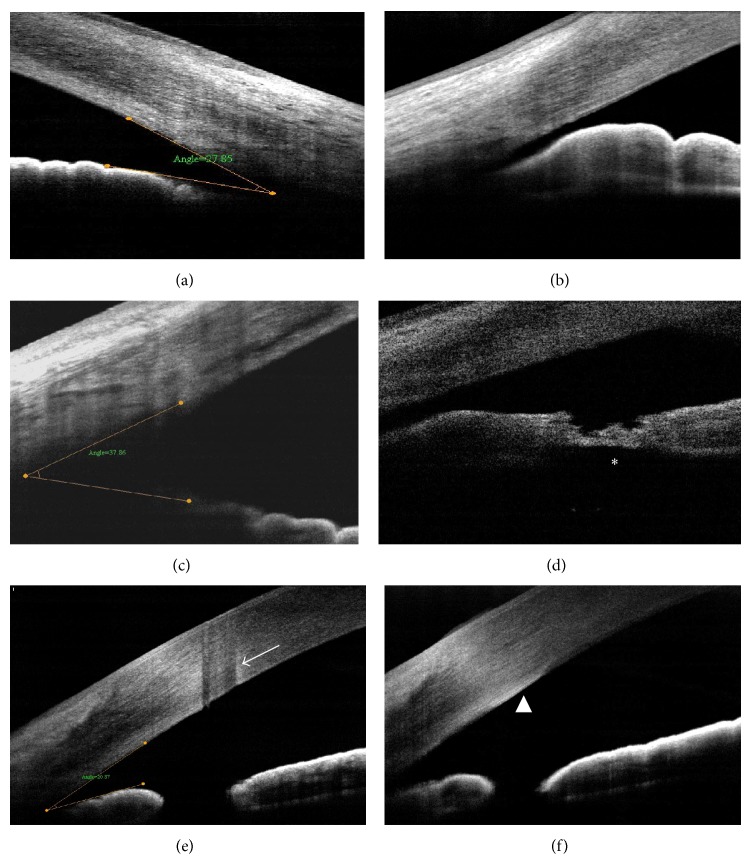
Angle assessment by AS-OCT. Narrow occludable angle (a), plateau iris configuration (b), open angle (c), incomplete laser iridotomy (d), stromal damage (e), and thickened Descemet's membrane after laser iridotomy (f).

Fourier domain OCT (FD-OCT), however, had limited visualizations for the ciliary sulcus and posterior border of the ciliary body in most of the cases because the iris pigment epithelium is not transparent with regard to infrared light, and poor definition of the scleral spur was also reported in approximately 25% of AS-OCT images [58] (Figure 11). Despite these disadvantages, OCT had additional merits, such as a noncontact-type performance without an artificial opening of the angle.

Angle assessment for glaucoma may be widely applicable in clinical situations: screening for angle closure [58], evaluation of the structural cause (including plateau iris configuration, pupillary block, and malignant glaucoma), evaluation of the efficacy of a laser procedure [56, 57] (patency, angle change, and concavity), and dynamic analysis of the iris configuration [59]. In the aspect of treatment outcomes, Lee et al. [57] reported that the ACA parameters changed significantly after laser iridotomy, but ACA remained unchanged in some narrow-angle eyes despite iridotomy.

Concerning the safety profile, laser iridotomy is a relatively safe procedure, but there is still a potential risk of corneal endothelial damage. Many proposed mechanisms were hypothesized: direct focal injury, thermal damage, mechanical shock wave, iris pigment dispersion, transient-raised intraocular pressure, inflammation, and breakdown of the blood-aqueous barrier [60]. To the best of the author's knowledge, however, there has been no case report of endothelial damage demonstrated by AS-OCT. The author presents a case with stromal damage and thickened Descemet's membrane accompanying endothelial damage after laser iridotomy (Figure 11).

Although angle assessment via AS-OCT was shown to have poor quality compared with UBM, AS-OCT may be helpful in various clinical situations in glaucoma patients because of its noncontact nature.

## 13. Glaucoma: Assessment of the Filtering Bleb and Tube

**Figure 12:**
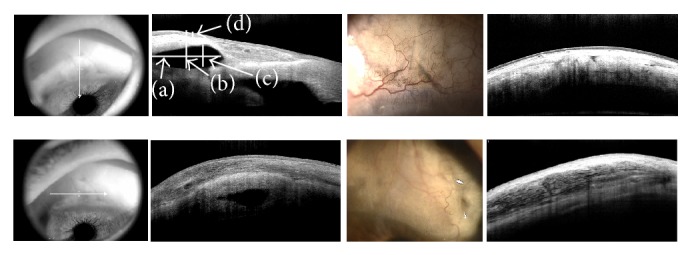
Anterior segment OCT images after trabeculectomies. Vertical and horizontal OCT sections (left column) demonstrate various parameters: internal cavity extent (a), bleb height (b), internal cavity height (c), and bleb wall thickness (d). Right upper images demonstrate thick bleb wall and high internal reflectivity suggesting bleb failure. Right lower images demonstrate relatively thin bleb wall and multiple-layer appearance with low internal reflectivity with good IOP control.

Although OCT was not developed to evaluate the filtering bleb, AS-OCT can visualize filtering blebs and can reveal the details of their morphology (Figure 12) [61]. Many articles have described the association of the bleb morphology and IOP control. Nakano et al. [62] predicted bleb failure based on the bleb wall uniformity in developing bleb after trabeculectomy. They reported that the multiple-layer appearance had a good bleb function after six months. Tominaga et al. [63] also stated that the low-reflectivity wall and the presence of episcleral fluid were associated with good IOP controls after trabeculectomy. Pfenninger et al. [64] demonstrated the correlation between the internal reflectivity of the fluid-filled cavity and IOP control after the recent trabeculectomy. The AS-OCT results, however, revealed no significant association between reflectivity and IOP control in Ahmed glaucoma valve (AGV) surgeries, and the maximum bleb wall was thinner in the surgical success group compared with the surgical failure group [65]. Recently, the 3D AS-OCT technique allows a detailed evaluation of the internal morphology of filtering blebs and precise identification of the filtration opening on the scleral flap margin after trabeculectomy [66].

**Figure 13:**
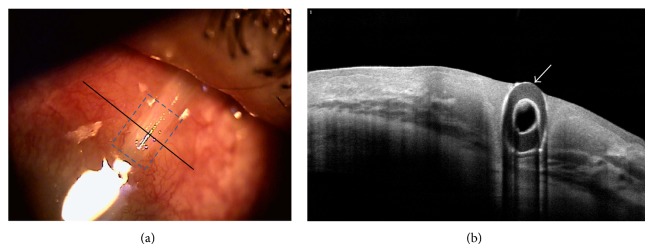
Tube erosion of glaucoma drainage device. Anterior segment optical coherence tomography image. Slit lamp examination revealed an extruded tube of glaucoma drainage device (GDD, Ahmed valve) with melting of scleral patch and conjunctival erosion. (a) Anterior segment optical coherence tomography (OCT) also displayed the GDD tube extrusion and loss of overlying conjunctiva (b).

In a clinical situation, AS-OCT was applicable for determining which blebs were suitable for needling [67] and which could be used to evaluate the bleb change after laser suture lysis [68]. Moreover, it provided detailed information when planning bleb revision surgery for overhanging filtering bleb [69]. In author's opinion, AS-OCT may also be helpful for distinguishing the “real” tube erosion of glaucoma implant surgery because of its noncontact nature (Figure 13).

In summary, the bleb wall anatomy is well assessed by AS-OCT while UBM is superior in evaluating a deep structure. Particularly, AS-OCT can be widely used in the early postoperative stage because of its noncontact characteristics [70].

## 14. Explanatory Text, Figure 13

A 64-year-old male with neovascular glaucoma secondary to proliferative diabetic retinopathy underwent glaucoma drainage device (GDD) implantation in his right eye. Insertion of an Ahmed glaucoma valve (Model FP7, New World Medical Inc., Rancho Cucamonga, CA) with a 4 × 4 mm sized overlying scleral patch graft was performed through a fornix-based conjunctiva approach.

Twelve months after surgery, overlying scleral patch graft was not visible. And three months later, he visited the author's clinic due to foreign body sensation, redness, and mild ocular tenderness for several days.

Slit-lamp examination demonstrated an extruded tube with melting of scleral patch (blue box) and conjunctival erosion. AS-OCT also showed the loss of conjunctival tissue overlying GDD tube. Arrow indicated the hyperreflective tear film on extruded tube. To the best of the author's knowledge, this is first photographic report on tube erosion of GDD demonstrated by AS-OCT.

## 15. Conclusions

In summary, AS-OCT can be widely applicable in various clinical diseases, including tear meniscus evaluation, ocular-surface disease, corneal dystrophies and stromal diseases, tissue change analysis after cataract and glaucoma surgery, and angle assessment. This noncontact-type technology with a high resolution and high reproducibility provides comprehensive and quantitative information.
